# Control of asthma triggers in indoor air with air cleaners: a modeling analysis

**DOI:** 10.1186/1476-069X-7-43

**Published:** 2008-08-06

**Authors:** Theodore A Myatt, Taeko Minegishi, Joseph G Allen, David L MacIntosh

**Affiliations:** 1 Environmental Health & Engineering, Inc., 117 Fourth Avenue, Needham, MA, 02494-2725, USA

## Abstract

**Background:**

Reducing exposure to environmental agents indoors shown to increase asthma symptoms or lead to asthma exacerbations is an important component of a strategy to manage asthma for individuals. Numerous investigations have demonstrated that portable air cleaning devices can reduce concentrations of asthma triggers in indoor air; however, their benefits for breathing problems have not always been reproducible. The potential exposure benefits of whole house high efficiency in-duct air cleaners for sensitive subpopulations have yet to be evaluated.

**Methods:**

We used an indoor air quality modeling system (CONTAM) developed by NIST to examine peak and time-integrated concentrations of common asthma triggers present in indoor air over a year as a function of natural ventilation, portable air cleaners, and forced air ventilation equipped with conventional and high efficiency filtration systems. Emission rates for asthma triggers were based on experimental studies published in the scientific literature.

**Results:**

Forced air systems with high efficiency filtration were found to provide the best control of asthma triggers: 30–55% lower cat allergen levels, 90–99% lower risk of respiratory infection through the inhalation route of exposure, 90–98% lower environmental tobacco smoke (ETS) levels, and 50–75% lower fungal spore levels than the other ventilation/filtration systems considered. These results indicate that the use of high efficiency in-duct air cleaners provide an effective means of controlling allergen levels not only in a single room, like a portable air cleaner, but the whole house.

**Conclusion:**

These findings are useful for evaluating potential benefits of high efficiency in-duct filtration systems for controlling exposure to asthma triggers indoors and for the design of trials of environmental interventions intended to evaluate their utility in practice.

## Background

Asthma is chronic inflammatory disorder of the airways that induces a range of sub-clinical and clinical effects including but not limited to hyperresponsiveness, airflow limitation, and respiratory symptoms. Approximately 6.7% of adults and 8.5% of children in the United States are reported to suffer from asthma with the greatest prevalence among non-Hispanic black and Hispanic children under 18 years of age [1]. Triggers of asthma exacerbation are varied and include viral infections, certain animal allergens and criteria air pollutants, mites, environmental tobacco smoke (ETS), mold, chemical irritants, and exercise in cold air [2]. Reducing exposure to environmental agents shown to increase asthma symptoms or lead to asthma exacerbations is an important component of a strategy to manage asthma for individuals [3].

Numerous investigations have demonstrated that indoor air cleaning devices can reduce concentrations of asthma triggers in indoor air [4-10]. Some studies have reported associations between use of air cleaners and improvements in respiratory symptoms and breathing problems for children and adults with asthma or persistent allergic rhinitis [11-14]. However, the benefits of air cleaners for breathing problems have not always been reproducible [14-19]. An expert panel recently determined that the evidence offered by health studies is not sufficient to conclude that operation of indoor air cleaning devices alleviates asthma symptoms or improves pulmonary function [14,18-20].

The heterogeneity in results of air cleaner intervention studies for asthma symptoms may reflect in part the limited efficacy of the portable air cleaners used to mitigate exposure to airborne asthma triggers. Portable air cleaners typically have flow rates of 170 – 340 cubic meters per hour (m^3^/hr) and removal efficiency for fine particle mass (PM_2.5_) of only about 70% because of bypass around their high efficiency particle arrestance (HEPA) filters [21]. For a typical U.S. home size of 450 m^3^, a 180 m^3^/hr portable device has a theoretical particle removal rate of approximately 0.4 per hour (hr^-1^), about the same as the air exchange rate for a closed home. Air flow rates through room filters must be equivalent to several air changes per hour in order to achieve substantial control of airborne particulate matter [4]. In contrast, whole house, high efficiency air cleaning systems that can provide clean air delivery rates up to 10 times greater than a portable air cleaner and particle removal rates of approximately 7 per hour are now available for residences [22]. The mitigation of asthma triggers in indoor air by these systems and potential health benefits for sensitive subpopulations have yet to be evaluated.

To address this knowledge gap, we used an indoor air quality modeling system to examine peak and time-integrated concentrations of fungal spores, environmental tobacco smoke, respiratory viruses, and cat allergen in indoor air associated with natural ventilation, portable air cleaners, and forced air ventilation equipped with conventional and high efficiency filtration systems. As part of the modeling, we simulated several conditions that correspond to asthma management guidance published by the American Lung Association and the National Institutes of Health.

## Methods

We used the CONTAM multi-zone indoor air quality model developed by the National Institute of Standards and Technology (NIST) to estimate indoor concentrations of indoor allergens and irritants associated with asthma [23]. Airflow among indoor and outdoor zones of the building (i.e. rooms and ambient air) in CONTAM occurs via flow paths such as doors, windows, and cracks. Inter-zonal flow is based on the empirical power law relationship between airflow and the pressure difference across a flow path. Simulation of a mechanical ventilation system in CONTAM also induces circulation of air in CONTAM. After airflow among zones is established, mass balance equations are used to calculate pollutant concentrations based on the sources and sinks in each zone. Each zone (i.e. rooms, hallways) is treated as a single node wherein the air has uniform, well-mixed conditions throughout. Performance evaluations of CONTAM have demonstrated that the model simulations of inter-zonal flow and air exchange rate are within 15% on average of corresponding values measured in a single-family home and test home, respectively [24-29].

Our analysis included two residential building templates developed by NIST, a two story detached home and a single story detached home. Single family detached homes represent over 60% of the total housing stock in the U.S. [30]. The floor areas for the single story and two story-building templates are 180 square meters (m^2^) and 276 m^2 ^respectively. See Additional files 1 and 2 for floor plans of the templates. The templates were based on the U.S. Census Bureau American Housing Survey [31] and the U.S. Department of Energy Residential Energy Consumption Survey [32] and were intended to represent typical U.S. residential building stock [33]. We modified the NIST templates to allow for natural ventilation and leakage through and around windows sized to 11.5% of the area of each wall [34].

Both residential templates were modeled with six different ventilation and filtration configurations (See Table 1). The first configuration was a home with natural ventilation (N) and no capacity for indoor air cleaning. The remaining configurations each employ a central forced air heating and cooling system with differing degrees of filtration including: a standard 1 inch media filter (C), a standard 5-inch media filter (C5), the 1-inch filter with one portable HEPA unit in a bedroom (C+1P), the 1-inch media filter with a portable HEPA unit in the bedroom and one in the living/family room (C+2P), and a high efficiency electrostatic air cleaner with HEPA-like removal efficiency for aerosols (HE).

**Table 1:** Ventilation/Filtration Configuration Information

Abbreviation	Description
N	Natural ventilation with no air cleaning capacity

Forced Air Systems	

C	Conventional 1-inch media filter (MERV 2)
C5	Standard 5 inch media filter. Based on Perfect Fit 5 inch media filter, Model BAYFTAH26M, Trane Residential Systems, Tyler, TX, USA (MERV 8)
HE	High Efficiency System – CleanEffects™ Model TFD235ALAH000AA, Trane Residential Systems, Tyler, TX, USA

Forced Air Systems plus Portable Air Cleaners	

C+1P	Conventional 1-inch filter plus portable HEPA filter devices. Flow characteristics based on Quiet Flo HEPA Air Purifier Model 20316, Hunter Fan Company, Memphis, TN, USA. Filtration capacity based on Chen et al. (2006).
C+2P	Conventional 1-inch filter plus 2 portable HEPA filters devices (See above)

Homes with central systems were assumed to have air-handling units (AHU) balanced to provide 0.18 m^3^/min/m^2 ^(0.6 cfm/ft^2^) of air to each room in the house. The duty schedule during heating and cooling periods was simulated with 1-hour resolution based on output from representative runs of the EnergyPlus Energy Simulation Software [35]. In general, the fraction of each hour devoted to forced air heating or cooling was proportional to the difference between ambient temperature and a set point of 22°C (72°F). Hourly duty schedules ranged from 4 minutes per hour during temperate periods to 38 minutes per hour during extreme summer periods and 52 min during extreme winter periods. In simulations with the C1 and C5 filters, a conventional AHU that operated only during periods of heating or cooling demand was used. In the simulations with the high efficiency electrostatic air cleaner, we modeled a modern AHU equipped with a variable speed fan that operates at full speed during periods of heating and cooling demand and at half-speed during all other times. Portable air cleaners were modeled as operating at 118 m^3^/hr for 24 hours per day. For the single story home, the return air duct AHU was located in the living room, for the two story home, there was a return in the hallway of both the first and second story. An air supply diffuser was located in each room of both housing templates.

For simulations of central forced air systems, removal efficiencies for in-duct air cleaners were based on particle size-specific results observed in our prior assessment of in-duct air cleaning technologies conducted in a fully instrumented test home [22]. In that work, we found that the removal efficiency of a polydisperse test dust achieved by in-duct devices (specifically, 1-inch, 5-inch, and high efficiency electrostatic) was approximately 10% lower than the rated efficiencies determined according to ASHRAE Method 52.2, an industry standard performance metric [36]. Through diagnostic testing, we determined that the difference between the rated and in-use performance was the result of bypass where 10% of the airflow through the AHU fan entered the AHU cabinet downstream of the filter bay.

For the portable air cleaners, removal efficiencies were based on studies conducted for the National Center of Energy Management and Building Technologies [21]. Similar to the whole house testing, Chen et al. found approximately 30% leakage in portable units and that none of the portable air cleaners reached HEPA-like filtration.

Meteorological information is used by CONTAM to simulate force convection, radiant leakage, and corresponding air exchange rates. We used year 2005 meteorological data, including hourly wind direction and speed, dry and wet bulb temperature, relative humidity, and cloud cover data, obtained from the National Weather Service for the Cincinnati, Ohio area (Cincinnati/Northern Kentucky International Airport). We chose this area because Cincinnati has four distinct seasons and differences in ventilation are expected to vary by climatic conditions.

Using a temperature-based probabilistic approach based on data from an EPA analysis [37], window and door opening schedules were generated that produced total ventilation rates for centrally and naturally ventilated periods consistent with corresponding air exchange rates determined from field campaigns reported elsewhere [38-40]. During periods in which the windows were open, 40% of the total window area was assumed to be open. The AHU duty schedule and the window schedules were linked so that the AHU was never running when the windows were open. The front door was set to a schedule of opening for 15 minutes five times each day. Particle-size specific deposition rates to indoor surfaces were based on research by Thatcher and colleagues [41]. Due to limitations of the model, deposition rates were assumed independent of air exchange rate and the AHU duty schedule.

A set of indoor allergens and irritants that can play a significant role in triggering asthma attacks was the focus of our analysis. Generation rates and particle size distributions of the contaminants were based on experimental data available in the published literature. Details regarding inputs to the model for the allergens and irritants are presented in Table 2.

**Table 2:** Model Inputs for Contaminant Emission Rates and Filtration Removal Efficiency Rates

Contaminant /Particle size	Emission Rate	Deposition Rate (hr^-1^)	1-inch (%)	5-inch (%)	High Efficiency (%)	Portable (%)
Cat Allergen^a^						

0.54	0.0688 μg/hr	0.052	2.5	29.2	90.7	71
0.875	0.0688 μg/hr	0.15	2.5	29.2	90.7	71
1.6	0.1376 μg/hr	0.35	20.7	47	91.8	71
2.7	0.5502 μg/hr	1	20.7	47	91.8	71
4	1.8895 μg/hr	2.2	55.3	77.8	96.5	72
5.25	2.0953 μg/hr	3.5	55.3	77.8	96.5	80
7.4	5.5885 μg/hr	6.5	74.3	86.9	98.4	80
9	10.899 μg/hr	10	74.3	86.9	98.4	80

ETS^b^						

0.0575	1.31 mg/cig	0.02	0	14.6	90.1	70
0.1475	2.84 mg/cig	0.005	0	14.6	90.1	70
0.31	2.84 mg/cig	0.018	0	14.6	90.1	70
0.71	1.31 mg/cig	0.08	2.5	29.2	90.7	71

Outdoor Fungal Spores						

2.5	NA	0.9	14	47	91.8	71

Virus^c^						

2.1	35.3 q/hr	0.6	14	47	91.8	71
4.5	29.4 q/hr	2.8	55	77.8	96.5	72
7.3	1.8 q/hr	6.5	73	86.9	98.4	80
9.4	0.5 q/hr	10	74	86.9	98.4	80

^a ^Between the hours of 7 am – 10 pm, the cat allergen concentration increases for 33% from the intermittent allergen release. Emission rates based on Custovic et al. [76].

^b ^A total of 8 cigarettes per day. Per cigarette emission rates (mg/cigarette) based on Klepeis et al. [46].

^c ^Emission rate of infectious doses (or quanta) per hour (q/hr) based on Liao et al. [56].

### Cat Allergen

Emission rates for cat allergen were based on studies that characterized the occurrence, suspension, and removal of cat allergen, *Fel d 1*, inside homes [7,42,43]. Based on findings from those studies, we chose to model generation of cat allergen with a constant and intermittent source. The constant source was used to represent *Fel d 1 *levels in air during quiescent periods. The intermittent source represented resuspension of cat allergen caused by certain activities such as vacuuming or sitting on a couch [10,44]. The intermittent source released a burst of allergen once an hour during typical waking hours, 7:00 AM – 10:00 PM. The constant generation source was located in all rooms of the house other than the bedrooms, while the burst source was released only in the main living space (i.e. living room for template 72 and family room for template 28). We omitted release of cat allergen in bedrooms in order to evaluate the extent to which allergen avoidance achieved by restricting cats from bedrooms as recommended by the NIH (2007), may be influenced by the use and efficacy of indoor air cleaning systems.

Aerosols that contain cat allergen range in aerodynamic diameter from less than 0.4 micrometers (μm) to greater than 9 μm [7,45]. Previous research has demonstrated removal of airborne cat allergen by portable air cleaners with HEPA filters [7]. For the electrostatic air cleaner, we assumed that the removal efficiency of cat allergen was equivalent to the particle-size specific performance observed for standard test dust and described elsewhere [22].

### Environmental Tobacco Smoke

Particle size information and emission rates for ETS were based on information reported from studies of cigarette smoke in experimental chambers [46]. The total particle mass released for each cigarette was 8.3 mg with a release rate of 1.3 mg/min. A recent national survey indicates that the average adult smoker in the United States consumes 15 cigarettes per day [47]. Taking into account waking hours spent at home [48], we modeled ETS emissions as cigarette consumption within the home twice in the morning hours and six times in the evening hours. All cigarettes were assumed to be smoked in the main living space (i.e. living room for template 72 and family room for template 28). Particle size for ETS has been reported to range from 0.05 μm to 0.71 μm [49]. Removal efficiency for ETS is one component of the industry standard method for determining and rating the performance of indoor air cleaning technologies [50].

### Outdoor Fungi

In contrast to the other asthma triggers that were modeled as indoor sources, we modeled indoor air concentrations of airborne fungi that result from penetration of mold spores in ambient air. To coincide with the meteorological data noted earlier, daily mold spore counts for February 14 to November 23, 2005 measured at the Hamilton County Environmental Services Office in Cincinnati were obtained from the Hamilton County Air Quality Management Division. The daily observations from Cincinnati are short-term samples collected with a Rotorod Sampler (Sampling Technology, Inc., Minnetonka, MN) and therefore do not reflect the temporal variability of spore concentrations that may occur over the course of each day. In the absence of more complete data, we assumed that concentrations within the day were constant for purposes of this analysis. The outdoor level of total fungal spores reported in the data for Cincinnati ranged between 32 and 7935 spores per cubic meter (spores/m^3^) with a geometric mean of 881 spores/m^3^. As expected, outdoor spore concentrations were highest in the summer and early fall months. The aerodynamic diameter size distribution for total spores is large, ranging from 1 to 40 μm. While the dominant fungal genera, *Cladosporium*, has a aerodynamic diameter slightly less than 2 μm [49], the other dominant types, basidiospores and ascospores have aerodynamic diameters on the order of 5 μm [51]. Fungal allergens are borne on spores larger than 2.5 μm as well as hyphael fragments and fragmented spores smaller than 2.5 μm. Because of the absence of information on fungal fragment levels in outdoor spore data for Cincinnati and the paucity of large spore types in the data, we established 2.5 μm as a reasonable central estimate of the aerodynamic diameter for fungi in this analysis.

### Respiratory Viruses

We modeled the release of two respiratory viruses, influenza virus and rhinovirus, because they have been implicated as triggers of asthma exacerbations and essential information is available on their transmission [52] and aerosol properties. While respiratory syncytial virus and other viruses have also been associated with asthma, a lack of key information on these organisms precluded their inclusion in this analysis. For our respiratory virus modeling we utilized the concept of infectious dose, referred to as quanta, as first described in 1955 [53] Estimates of quanta generation rates from an infectious person are based on analyses of outbreaks of infectious diseases as described elsewhere [54,55]. The greater the quanta generation rate the more infectious the organism. Estimates for influenza, a virus that can spread rapidly, are on the order of 15 to 128 quanta per hour [54,56]. Organisms with slower spreading infections, like rhinovirus and tuberculosis have generation rates on the order of 1 to 10 quanta per hour [54]. For this analysis, we assumed the approximate mid-point of published quanta generation rates for influenza and rhinovirus, 67 q/hr and 5 q/hr, respectively.

We also assumed the quanta were evenly distributed among the particles released during a sneeze. The removal processes are based on the particle sizes of the quanta released. We based the particle size distribution on experimental studies of particles emitted during sneezes and coughs conducted in the 1940s and 1960s [57,58] and recently re-analyzed [59]. To establish removal efficiency for respiratory virus achieved by the in-duct media filters, we relied upon size-specific results observed in our test home [22]. For the in-duct electrostatic air cleaner, the removal efficiency was based on laboratory studies in which a suspension of live influenza A virus, PR-8 strain (Advanced Biotechnology, Inc., MD) in phosphate buffered saline was aerosolized within a ventilation duct using a 6-jet Collison nebulizer. Aerosol samples were obtained on Teflo filters (Millipore Corporation, Bedford, MA) in triplicate upstream and downstream of the electrostatic air cleaner on three days. The samples were extracted and assayed for influenza by quantitative polymerase chain reaction (qPCR) following procedures described by Van Elden et al. [60]. The average removal efficiency from the tests was greater than 99% with more precise quantitation limited by the sensitivity of the assay. The removal efficiencies obtained from the laboratory studies were coupled with AHU bypass information for use in the model. Details of this novel application of qPCR will be published elsewhere.

Output from the IAQ model for respiratory virus was expressed as quanta per cubic meter (q/m^3^) of indoor air. We used a modified Wells-Riley equation [61] to estimate the risk of infection based on the concentration of quanta in the room from the model output coupled with conventional central estimates of exposure duration and a breathing rate of 0.48 m^3^/hr published in a widely used compilation of exposure factors [62]. We used the results to analyze the risk of infection for an individual when (1) spending time in the same room as an infectious individual, (2) spending time in an adjacent room, and (3) occupying other rooms in the house when an infected individual is either in a bedroom or in the living room of the home.

## Results

Air exchange rate (AER) is an influential determinant of indoor air quality and hence is a primary output from the CONTAM model. The distributions of 24-hour average AER across the year for the two templates with both natural and forced air ventilation systems are summarized in Table 3. The mean and median AER for the natural ventilation configuration were approximately twice those in the forced air configuration due to the increased use of windows during warm weather. AER was lower in the newer home (DH28) than the older home (DH72) which reflects differences in leakage rates between the two homes. With the exception of differences in AER, the modeling results were similar for the two home templates. Therefore, we chose to report only the results from the newer two-story home (DH28).

**Table 3:** Distribution of simulated 24-hour average air exchange rates for homes with and without forced air ventilation systems.

Ventilation/Filtration	House Template	Mean	Std Dev	Percentiles				
				
				5%	25%	50%	75%	95%
Natural	DH28	3.7	5.0	0.1	0.2	0.2	6.8	13.0
Forced Air		1.8	3.6	0.1	0.1	0.2	0.9	10.9
Natural	DH72	3.0	3.9	0.2	0.4	0.5	5.1	10.6
Forced Air		1.6	2.9	0.1	0.3	0.4	0.7	8.7

DH28 Two story detached home

DH72 Single story detached home

### Cat Allergen

**Figure 1:**
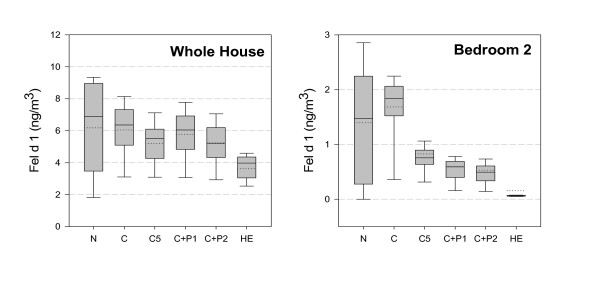
**Comparison of Hourly Fel d 1 allergen concentrations by filtration configuration for (1A) the whole house average and (1B) bedroom 2**.

The distribution of hourly average concentrations for airborne cat allergen throughout the home for each of the six ventilation configurations is summarized in Figure 1A. When operating a high efficiency device, the median allergen concentration (4.0 ng/m^3^) was 46% lower when compared to conventional filtration (6.4 ng/m^3^). The next best performance was achieved by two systems – the in-duct 5-inch media filter (C5) and a portable air cleaner in the same room as the intermittent release of allergen (i.e. C+2P). Nominally, peak concentrations were best mitigated by the high efficiency in-duct device (86 ng/m^3^), although the difference in comparison to peaks associated with the other air cleaning approaches (approximately 100 ng/m^3^) may not be substantive relative to uncertainties in the modeling analysis. To evaluate the effectiveness of the ventilation configurations at limiting transfer of allergen to bedrooms, all airborne releases of cat allergen in our model occurred outside of the bedrooms. In the bedroom, allergen levels were lower than the whole house average for all configurations, with the high efficiency in-duct filtration performing best at minimizing the transfer of allergen into the bedroom (See Figure 1B).

### Environmental Tobacco Smoke

**Figure 2:**
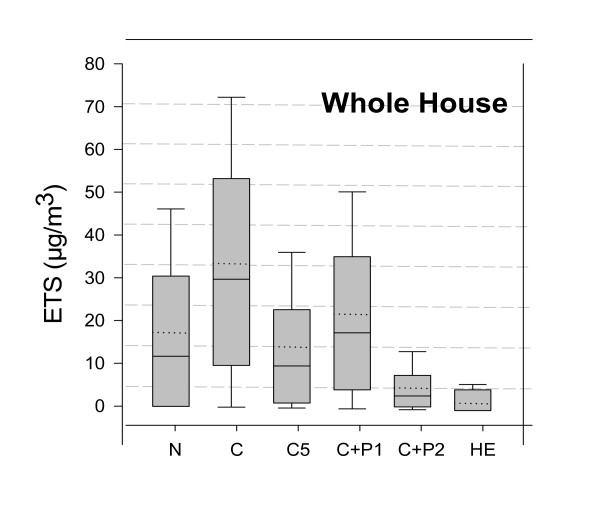
Comparison of Hourly ETS concentrations by filtration configuration.

Modeled whole house concentrations of ETS were strongly influenced by use of air cleaners as illustrated by the distribution of hourly average concentrations estimated across the year (Figure 2). The greatest mitigation of ETS was achieved by the high efficiency in-duct device (median <0.01 μg/m^3^), followed by use of a portable air cleaner in the same room as the smoker (median 3.2 μg/m^3^), the pleated in-duct media filter (median 9.8 μg/m^3^), one portable air cleaner in a bedroom (median 17.8 μg/m^3^), and a conventional in-duct filter (median 29.9 μg/m^3^). Simulation of a home with natural ventilation yielded hourly average ETS concentrations that were similar to the C5 simulation, probably because of the higher AER throughout the year for a home without forced air conditioning.

**Figure 3:**
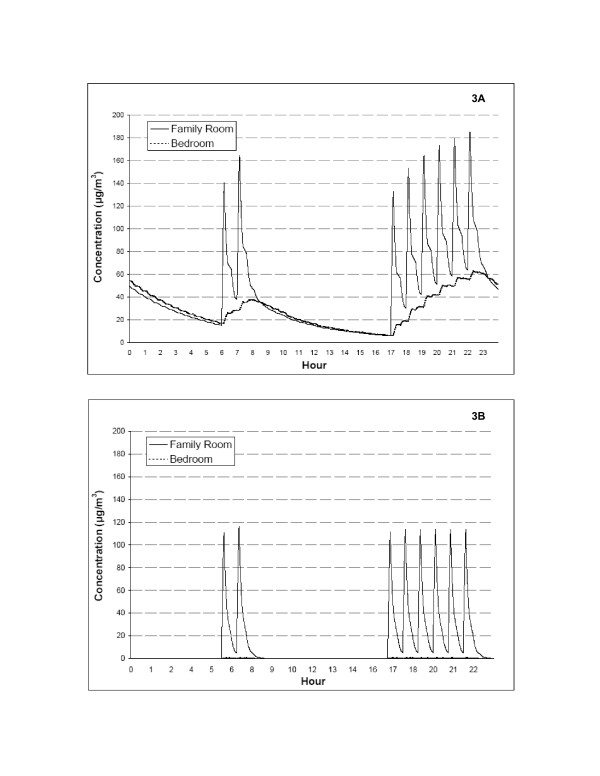
Comparison of 24-hour environmental tobacco smoke (ETS) concentrations in the living room and bedroom between the conventional filter (3A) and the high-efficiency filter (3B) for February 1.

The effect of high efficiency in-duct filtration on peak and short-term time-weighted averaged levels of ETS is depicted in Figure 3A and 3B for a typical 24-hour period (February 1) that had eight smoking events in the living room. For a home with conventional in-duct filtration, each cigarette smoked is associated with a peak concentration of approximately 80 μg/m^3 ^and a subsequent exposure period of at least 8 hours when windows are closed. In contrast, peak ETS concentrations per cigarette during model runs with the high efficiency in-duct device were about 40 μg/m^3^. First-order removal rates for ETS calculated for the conventional and high efficiency in-duct filtration conditions were 0.008 min^-1 ^and 0.049 min^-1^. Use of the high efficiency in-duct device also substantially limited the distribution of the contaminant into other rooms of the home such as the bedroom.

### Outdoor Fungi

The highest indoor/outdoor ratios for spore concentrations were in the summer and fall months, probably due to the higher AER associated with open windows during those seasons. When averaged over the period for which fungal spore data were available, the indoor/outdoor ratio was highest for the natural ventilation configuration and lowest for the in-duct high efficiency configuration (Table 4). Whole house indoor spore concentrations for the in-duct high efficiency configuration were less than one-half the levels in the conventional configuration and more than eight times lower than the mean outdoor level. Even in the bedroom where the portable air cleaner was located, the in-duct high efficiency achieved lower spore levels.

**Table 4:** Geometric Mean (Geometric Standard Deviation) of Indoor/Outdoor Ratios and Indoor Spore Concentrations by Ventilation/Filtration Type

Ventilation on/Filtration	I/O Ratio	Whole House (spores/m^3^)	Bedroom 2^a^ (spores/m^3^)
N	0.34 (2.6)	303 (7.0)	238 (9.4)
C	0.16 (2.7)	141 (5.8)	131 (6.3)
C5	0.13 (3.1)	111 (6.7)	97 (8.0)
C+1P	0.14 (2.9)	128 (6.0)	54 (8.5)
C+2P	0.14 (2.9)	119 (6.2)	52 (8.8)
HE	0.07 (4.1)	57 (8.3)	41 (13.0)

^a^Bedroom 2 contains a portable air cleaner

### Respiratory Viruses

For the virus results, we limited the analysis to December through March to reflect the cold and flu season in the United States. The median AER for this period was 25% lower for the naturally ventilated configuration and essentially unchanged for the mechanically ventilated homes in comparison to the remainder of the year. For this period, we examined the extent to which the risk of infection by either influenza or rhinovirus is modified by the use of an air cleaner for three common scenarios where a healthy individual and infectious individual cohabitate.

In the first scenario, a healthy individual, perhaps a caregiver, spends one hour in the bedroom of an individual infected with influenza. In this case, the use of a portable air cleaner in the room with the infectious individual limits the average risk of infection to less than one-half the risk when conventional filtration is used (Table 5). The high efficiency in-duct system provides the next lowest average risk of infection, followed by the conventional and pleated filter in-duct systems. The risk of infection is lowered for each of the in-duct and portable air cleaner configurations in comparison to natural ventilation

**Table 5:** Mean (Standard deviation) percent risk of infection during three exposure scenarios

Scenario	1	2	3	
Ventilation /Filtration	Risk of influenza infection for a one hour exposure in the bedroom with individual infected with influenza	Risk of influenza infection from 12 hour exposure in adjacent bedroom	Risk of infection during 5 day infectious period while infected individual in bedroom for 1/2 the day and the family room for the 1/2 the day^a^	
			
			Influenza	Rhinovirus

N	36 (7.9)	0.6 (1.3)	17.1 (2.4)	1.4 (0.2)
C	18 (3.4)	16.1 (1.7)	70.0 (1.6)	8.6 (0.4)
C5	17 (3.4)	6.7 (1.0)	36.6 (1.8)	3.4 (0.2)
C+1P	7 (0.8)	5.9 (0.7)	51.9 (1.7)	5.3 (0.2)
C+2P	7 (0.8)	5.4 (0.6)	33.7 (2.2)	3.0 (0.2)
HE	13 (1.5)	0.6 (0.1)	3.9 (0.2)	0.3 (0.01)

^a ^Assumes that that occupant is in the home 68.7% of the time based on Klepeis et al. [48]

In the second scenario, we evaluated the risk of infection for a person who spends 12 hours in a bedroom adjacent to a second bedroom occupied by an individual infected with influenza. This scenario is representative of many residential configurations including children who normally sleep in separate bedrooms or two children who normally share a bedroom but are separated temporarily when one of them has a chest cold. In this scenario, the risk of influenza infection for a 12-hour exposure for an occupant in the adjacent bedroom was approximately 16% with conventional filtration, 5% for the configurations with a portable air cleaner in the bedroom and 0.6% with the high efficiency filtration (See Table 5).

For the third scenario, we estimated the risk of infection from an individual who remains in the home over the course of a five-day infectious period. We assumed that the infectious individual spent one-half of their time in the bedroom and the other half in the family room, while a healthy individual spent 69% of the corresponding time indoors at home [48] during which they were exposed to the house-wide average concentration of quanta in air. For this scenario, the risk of infection by influenza was greater than 30% in the ventilation configuration with a portable air cleaner in both of the two rooms frequented by the infectious individual (Table 5). In comparison, the risk of infection was 17% for the natural ventilation configuration and less than 4% for the high efficiency in-duct system. The former probably reflects a relatively slow rate of inter-zonal transfer and the latter reflects the comparatively high flow rate and removal efficiency of the in-duct system.

## Discussion

Several studies have assessed the use of air cleaners for reducing indoor air concentrations of chemical and biological materials that exacerbate asthma. In these studies, the air cleaning intervention was typically a portable air cleaner sized for a single room of typical size in a residence. Although based on modeling rather than measurements, our analysis indicates that certain air cleaning configurations can mitigate indoor air concentrations of some common asthma triggers more effectively on average than air cleaning achieved by the type of portable filtration devices evaluated previously as well as by conventional in-duct filtration.

Prior performance evaluations of CONTAM demonstrate that the model provides a reasonable degree of accuracy for the types of indoor air quality simulations upon which our analyses rely. Inter-zonal airflow predictions from CONTAM simulations of a single story home were within 15% of corresponding measured values [29]. Similarly, air exchange rates for a single room building predicted with CONTAM were within 5% of measured levels [24]. In a related analysis, the correlation between predicted and observed concentrations of a conservative gas ranged from 0.95 to 0.998 during six tests within a single room test home [25]. In a tracer gas study conducted in a multi-room occupied townhouse, gas concentrations predicted by the model were within 25% of measured concentrations [26]. Finally, measured and predicted 24-hour average concentrations of 0.3 to 5 μm particles in a single room building were within 30% of each other [24].

Particle removal efficiencies for air cleaning systems considered in this analysis were derived from empirical data obtained from test homes or test chambers [21,22]. Removal efficiencies for the portable air cleaners were based on chamber studies of four different devices that all claimed to have HEPA filters but whose efficacy under controlled conditions was low compared to HEPA standards [21]. If we had assumed that the portable air cleaners had removal efficiencies approaching those of HEPA filters, those systems would have compared more favorably to the other devices for the rooms of the homes in which they were located. Whole house comparisons of portable and in-duct systems are unlikely to have been changed substantially if we had assumed a higher aerosol removal efficiency for the portable devices.

In terms of controlling residence-wide concentrations of cat allergen, ETS, respiratory viruses, and mold spores in indoor air, use of a high efficiency in-duct air cleaner as part of a forced air ventilation system yielded the greatest benefit, followed by multiple portable air cleaners in conjunction with conventional in-duct filtration. The greatest benefit of air cleaning systems over conventional in-duct filtration was observed for ETS, probably because of its sub-micron size distribution and the correspondingly low rate of deposition to surfaces. The extent to which these findings can be generalized to other constituents of indoor air depends upon their similarity in terms of emission profiles and aerodynamic characteristics. Other important indoor allergens such as dust mite and cockroach that have been shown to be associated with relatively particles are unlikely to be represented accurately by our results for cat allergen, ETS, viruses, and fungal spores.

Consistent with results from our evaluation of air cleaners in a test home [22], the whole house performance of each system was directly related to its clean air delivery rate (CADR), the product of air flow rate and removal efficiency. This analysis focused on single family detached homes, however we anticipate that the findings are applicable to multi-family and attached homes as well. Various types of housing stock may differ systematically in terms of air exchange rate because of variation in construction practices, exterior surface area-to-volume ratios, and other factors. Particle deposition has been reported to be positively associated with air exchange rate due to increased turbulence of indoor air [63,64]. Because of modeling constraints, we assumed that particle deposition rates were independent of air exchange rate. This simplifying assumption is unlikely to be a substantial contributor to uncertainty in our results because the range of turbulence-induced deposition rates reported for respirable-sized aerosols is small in comparison to differences in performance among air cleaning devices indicated by our analysis.

In terms of controlling the contaminant concentrations in a single room, the location of the contaminant source is important. If the contaminant source was in the family room of the home and therefore near a central return, as was the case for the allergen and ETS modeling, the high efficiency in-duct filtration was superior to all configurations including those with a portable air cleaner in the room. Similarly, if the source is outdoors, as was the case with the fungal spore modeling, the high efficiency in-duct filtration was superior. Conversely, when the source was in a location away from a central return, like a bedroom, as was the case for the one-hour influenza scenario, operation of a portable air cleaner in the room was the most effective air cleaning configuration. We anticipate that these results for cat allergen, ETS, and virus are reasonably representative of emissions of other respirable-sized aerosols from indoor sources including fungal spores that may be released from surfaces as a result of mechanical forces.

The utility of the modeling results presented is related primarily to relative differences between the air cleaning systems included in this assessment. If reasonable however, the absolute levels are also of interest for consideration of potential air quality and exposure benefits afforded by indoor air cleaning systems. To assess the accuracy of the model results, we compared the predicted concentrations to measurements from studies that quantified residential airborne levels of animal allergens [5,7,10], ETS [4,65], or fungal spores [6,66]. Several of the studies evaluated the effectiveness of portable air cleaners with HEPA filters which allows us to compare our modeled results to not only the reported levels, but also to the changes in contaminant concentrations associated with use of portable air cleaners. Other studies were designed to measure typical residential contaminant concentrations, both with and without a source present. Data from those investigations provide a reasonable benchmark for our modeled results under typical ventilation configurations.

The relative differences among the ventilation configurations that we considered are similar to the reductions observed in intervention studies designed to evaluate the effectiveness of portable air cleaners. In a study of dog allergen, airborne levels in two rooms with portable air cleaners were reduced to 25% of the baseline allergen level [5]; similar to the difference in modeled cat allergen concentrations for the bedroom when the portable air cleaner was introduced. In a study of portable air cleaner efficacy in four homes with smokers, PM concentrations in the living room were reduced by 30–70% with the use of a portable air cleaner [4]. Our modeling yielded similar reductions in ETS when comparing the conventional filtration to the ventilation configurations with portable air cleaners. In a study designed to evaluate the utility of portable air cleaners for controlling fungal spore concentrations, the intervention effectively reduced spore levels in a bedroom of a residence, however the air cleaner worked best when the bedroom door was closed [6].

When comparing absolute levels of contaminants in the home, the modeled results for the conventional and natural ventilation configurations compare well with values reported in the literature. Our modeled cat allergen concentrations with conventional filtration are similar to concentrations reported in a study of 75 homes with cats in Britain [7] and the levels during our intermittent release of allergen is similar to measured values during periods of disruptions such as vacuuming [10,67]. In a study of homes in six U.S. cities, the authors calculated that smoking one pack of cigarettes daily contributed 20 μg/m^3 ^to the 24 average hour indoor particle concentration [65], which is similar to our modeled ETS concentrations for the conventional and natural ventilation configurations. For fungal spores, our modeled indoor/outdoor ratios for the conventional and natural ventilation configurations are similar to the ratio of 0.32 for total spores reported in a study conducted in six homes in the Cincinnati area [66].

Results from a controlled study of rhinovirus transmission provide a reasonable comparison for evaluating the accuracy of our modeled likelihood of infection. In the experimental study, groups of eight students with active rhinovirus infection spent 12-hours in a room with 12 susceptible students and followed a protocol designed to allow transmission of an infectious dose only by inhalation [52]. The resulting risk of infection from this study was 61%. While AER or filtration characteristics were not reported for this study, we assumed that the room was either naturally ventilated or had conventional filtration. Our modeled scenario with one infectious individual in a room of approximately one-half the size of the experimental room resulted in an average risk of infection with influenza of 33.6% and 16.5% with natural and conventional filtration, respectively. If the modeling were conducted with four infectious individuals in the smaller bedroom to more closely mimic the conditions of the experimental study, the risk of modeled infection would rise to a level similar to that observed in the experimental study.

We relied upon the concept of quanta generation to estimate the probability of acquiring an infection through the airborne route, using the Wells-Riley equation [61]. The Wells-Riley equation and modifications of the equation have been used by researchers to estimate the risk of airborne transmission of an infection for a variety of organisms including measles [61], influenza [54], rhinovirus [54,68], severe acute respiratory syndrome (SARS) [69], and tuberculosis [55]. The Wells-Riley equation only estimates the risk of transmission for the inhalation route of exposure. Organisms like rhinovirus and influenza can be transmitted by other routes of exposure such as direct contact, although the relative importance of the respective routes of exposure is not well understood. The ability of various indoor air cleaning configurations to influence virus transmission through surface-mediated pathways remains to be determined. Consideration of virus transmission via surfaces and other pathways is unlikely to influence our findings for modification of the risk of infection through inhalation because of different ventilation and air cleaning configurations.

While the Wells-Riley equation accounts for the ventilation rate of the indoor space of interest to calculate the quanta concentration, it does not, as discussed recently [70], explicitly account for other removal processes such as deposition to surfaces, filtration, and loss of infectiousness in the air. However, quanta generation rates are typically based on disease outbreak data, and therefore inherently account for these processes. Our modeling accounted for deposition and filtration, but not loss of infectiousness. Some data suggest that virus die-off is a slow process that can occur over several days at temperature and humidity levels typical of indoor environments [71]. Therefore, not explicitly controlling die-off is unlikely to influence our results substantively. Including removal mechanisms in our model along with the estimates of virus emissions in units of quanta may have resulted in double counting for removal by filtration and deposition. Therefore, our results may underestimate the actual risk of infection. To evaluate the impact of potential double counting for deposition, we conducted model runs without a deposition rate for virus. In these models, the risk of infection increased approximately 30 to 50% depending on the filtration type. Regardless, our analysis was designed to primarily evaluate the differences in ventilation configurations and the differences between these configurations would not be changed by under or over estimating the risk of infection.

While a number of intervention studies clearly demonstrate exposure reductions attributable to the use of portable air cleaners, associated improvements in health have been more difficult to demonstrate. Some air cleaning interventions have yielded improvements in respiratory symptoms and breathing problems for children and adults with asthma or persistent allergic rhinitis [11-13,72]; however, the results of these studies have not always been reproducible [14-18,73]. One explanation for the lack of reproducible results could be that portable air cleaners used in these studies have not effectively reduced personal exposure. Our modeling demonstrates that while the use of a portable air cleaner will provide exposure benefits in the room it is located, concentrations of common asthma triggers throughout the residence, and corresponding personal exposures, are not likely to be mitigated. Our modeling analysis indicate that high efficiency in-duct air cleaning systems would yield a more substantial reduction in personal exposure that the portable air cleaners used in intervention studies published to date. Potential benefits of these systems for personal exposure could be evaluated following methods employed in a study of personal exposure to cat allergen [74].

An Expert Panel convened by the NIH recommended asthmatics with pet allergies that are not willing to part with their pets keep the pet out of the asthmatic's bedroom as one part of an asthma management strategy. Additionally, the National Environmental Education & Training Foundation (NEETF) recommends that the use of portable air cleaners in bedrooms of asthmatics [75]. While the use of portable air cleaners in the bedroom prove to be beneficial in our modeling, the results indicate that the use of high efficiency in-duct air cleaners provide an more effective means of controlling allergen levels not only in a single room, but the whole house.

## Conclusion

The modeling results from this study demonstrate that properly maintained forced air systems with a high efficiency aerosol removal system are expected to provide the best control of the indoor exposure to common asthma triggers such as cat allergen, ETS, fungal spores and respiratory viruses. The modeling results also showed that the potential efficacy of avoidance strategies recommended for asthmatics by the American Lung Association and the National Institutes of Health may be enhanced by the use of certain indoor air cleaning systems.

## List of Abbreviations

ETS: Environmental tobacco smoke; PM_2.5: _Particles less than 2.5 microns; HEPA: High efficiency particle arrestance; NIST: National Institute of Standards and Technology; AHU: Air-handling units; AER: Air exchange rates; SARS: Severe acute respiratory syndrome; CADR: Clean air delivery rate; qPCR: Quantitative polymerase chain reaction. 

## Competing interests

Funding for this research was provided by Trane Residential Systems, Inc., Tyler, TX and Environmental Health & Engineering, Inc., Needham, MA.

## Authors' contributions

TAM conceived of the study, and participated in its design and coordination and helped to draft the manuscript. TM carried out the modeling efforts. JA carried out the data analysis. DLM participated in the design of the study and drafted the manuscript. All authors read and approved the final manuscript.

## Supplementary Material

Additional file 1 Two-Story Home Floorplan (DH28). Click here for file

Additional file 2 One-Story Home Floorplan (DH72). Click here for file
