# Supplementary figures and images for: Control of asthma triggers in indoor air with air cleaners: a modeling analysis
Source: Environ Health. 2008 Aug 6;7:43. doi: 10.1186/1476-069X-7-43 (PMC2543006; doi:10.1186/1476-069X-7-43)

MODEL DH-F (2)  
2966 S.F.  
2-STORY  
4 BR, 3-1/2 BATH, 4 ADD'L ROOMS

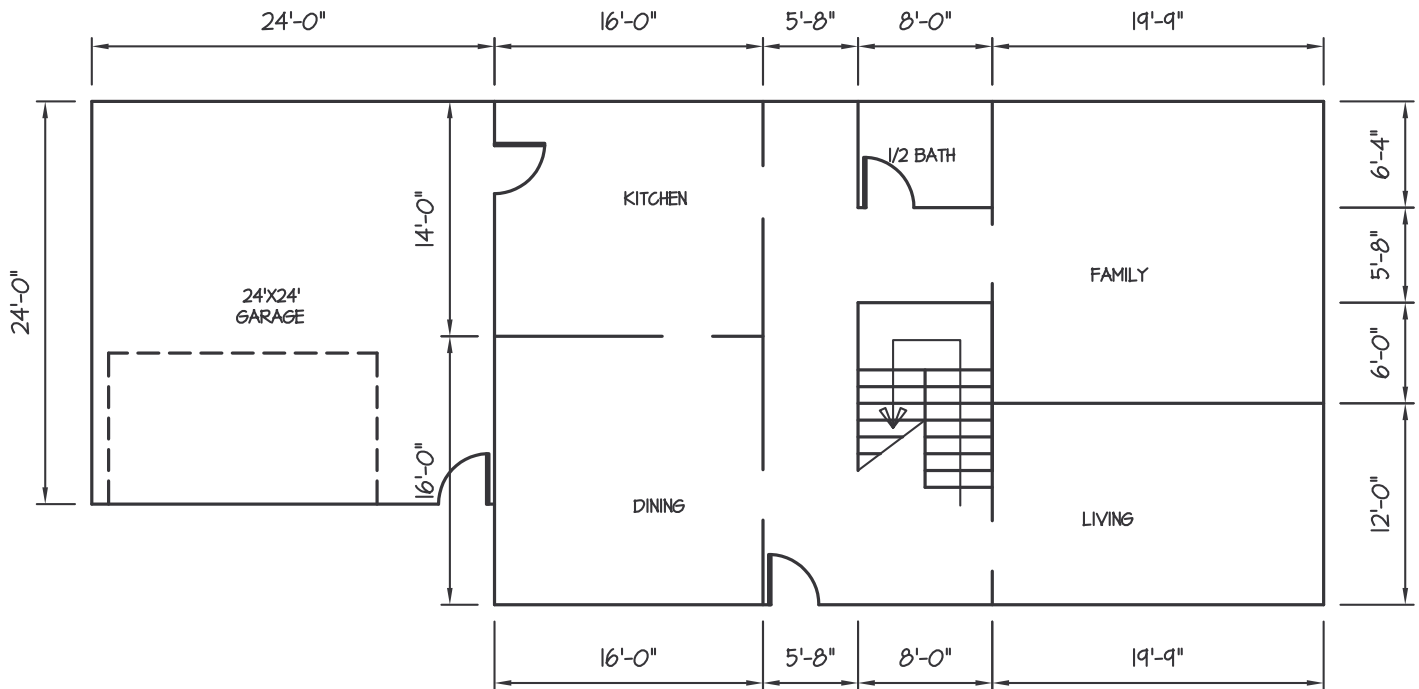

FIRST FLOOR

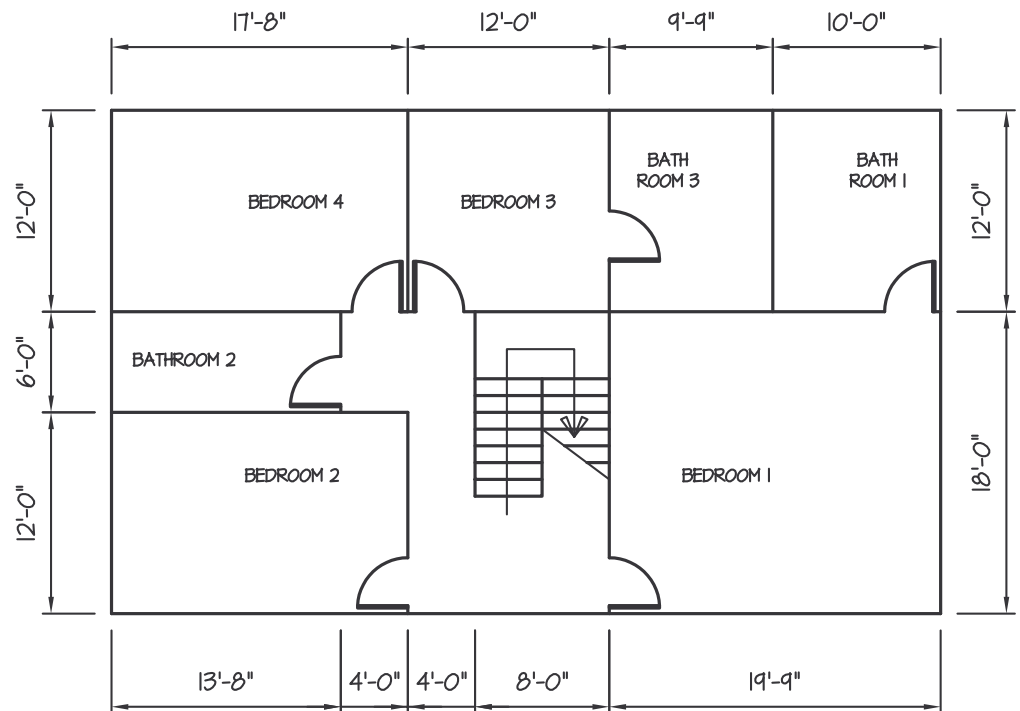

SECOND FLOOR

Supplement: Additional file 1 — Two-Story Home Floorplan (DH28). [file 1476-069X-7-43-S1.pdf]

MODEL DH-B (3)

1942 S.F.

1-STORY

3 BR, 2 BATH, 4 ADD'L ROOMS

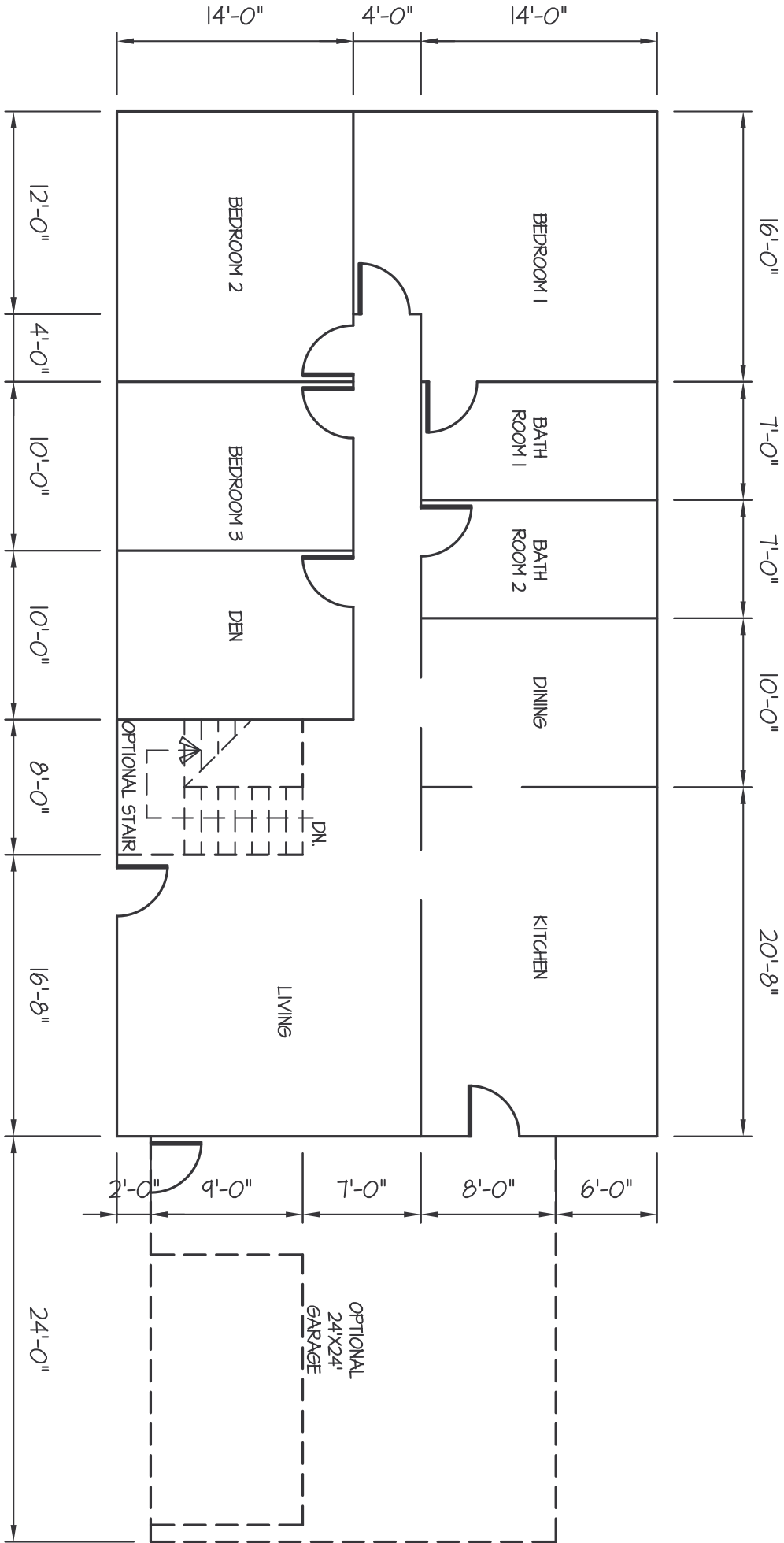

Supplement: Additional file 2 — One-Story Home Floorplan (DH72). [file 1476-069X-7-43-S2.pdf]
